# CNN-GRU-KAN: A Novel Multi-Branch Framework for Parkinson’s Disease Detection Based on Gait Classification

**DOI:** 10.3390/bioengineering13080905

**Published:** 2026-08-10

**Authors:** Xingkai Fu, Minlan Jiang, Mohammed A. A. Al-qaness

**Affiliations:** 1 College of Physics and Electronic Information Engineering, Zhejiang Normal University, Jinhua 321004, China; xingkai_fu@zjnu.edu.cn (X.F.); xx99@zjnu.cn (M.J.); 2 College of Engineering and Information Technology, Emirates International University, Sana’a 16881, Yemen

**Keywords:** Parkinson’s disease, health informatics, Kolmogorov–Arnold networks, gait classification, deep learning

## Abstract

Parkinson’s disease (PD) diagnosis and severity assessment increasingly rely on gait analysis. To capture complex gait dynamics from limited sensor data, we propose a novel multi-branch framework integrating a one-dimensional convolutional neural network (1D-CNN), a bidirectional gated recurrent unit (Bi-GRU), and Kolmogorov–Arnold networks (KANs) (CNN-GRU-KAN) utilizing 18-channel vertical ground reaction force (VGRF) signals. Each module serves a distinct clinical purpose: the 1D-CNN branch with squeeze-and-excitation (SE) attention extracts localized spatial plantar pressure patterns, while the Bi-GRU branch with temporal attention captures long-range rhythm abnormalities. Crucially, the KAN serves as the classification head. By utilizing learnable B-spline functions instead of traditional fixed activations, KAN adaptively models the highly non-linear boundaries between healthy controls and varying PD severities, effectively mitigating overfitting. Under rigorous subject-independent cross-validation, our model achieves 98.43% accuracy for binary PD detection and 93.46% for five-class UPDRS severity grading. These results highlight the framework’s strong potential for low-cost, unobtrusive clinical tracking and home monitoring.

## 1. Introduction

Parkinson’s disease (PD) is a prevalent neurodegenerative disorder in which movement symptoms, including resting tremor, muscular stiffness, bradykinesia, postural instability, and gait disturbance, are frequently observed [1,2]. The disease markedly affects motor functioning and overall life quality [3]. Gait disturbance represents an early, prototypical manifestation of PD and holds significant clinical importance in disease diagnosis and progression evaluation [4,5]. Recent advances in wearable devices and sensor technologies have made automated gait analysis based on gait data an important research direction for auxiliary PD diagnosis [6,7].

In current clinical practice, the early diagnosis of PD primarily relies on empirical judgment by physicians. This typically involves a comprehensive assessment based on medical history, neurological examination, response to dopaminergic medications [8], and rating scales such as MDS-unified Parkinson’s disease rating scale (MDS-UPDRS) [9] and that of Hoehn and Yahr [10]. However, early PD signs are often subtle and may overlap with age-related changes, drug-induced Parkinsonism, or essential tremors. These assessments are susceptible to consultation time, evaluation environment, and medication state (on/off), leading to considerable subjectivity and inter-rater variability [11]. Additionally, auxiliary techniques such as functional neuroimaging are costly, lack accessibility, and are difficult to routinely implement, thus limiting their use for widespread screening and long-term monitoring [12,13]. Consequently, there is a growing clinical need for objectively quantifiable, reproducible, low-cost, and continuously monitorable digital biomarkers and automated diagnostic tools [14,15].

The rapid advancement of wearable sensor technology in recent years has facilitated the convenient acquisition of data on daily gait and motor behavior [16]. Signals such as plantar vertical ground reaction force (VGRF) [17,18], inertial measurement unit (IMU) data [19], and video-based motion sequences can finely characterize spatial-temporal gait abnormalities associated with PD [14]. Compared with conventional approaches that depend on manually designed features, deep architectures automatically derive discriminative spatio-temporal features under a holistic learning paradigm, thereby enhancing accuracy and cross-cohort generalization in early identification and stage estimation [20]. Therefore, building robust deep learning models based on objective gait signals holds promise for complementing clinical expertise and may provide a scalable technical pathway for early screening, quantification of disease progression, and home-based monitoring of PD [21,22].

Deep learning has been widely used in gait analysis, improving recognition accuracy [23,24,25,26]. However, single deep learning models often fail to handle both spatial and temporal correlations within gait data simultaneously, and their generalization and robustness can be further enhanced. Multi-branch fusion architectures offer a novel solution. By integrating a convolutional neural network (CNN) and a bidirectional gated recurrent unit (Bi-GRU), they can effectively mine multi-dimensional features of gait data, improving the model’s expression ability. In attention mechanisms like the squeeze-and-excitation (SE) module, the model’s focus on key information is strengthened, thus improving recognition performance. Moreover, replacing traditional fully connected layers with Liu et al. [27]’s Kolmogorov–Arnold network (KAN), which shows stronger non-linear modeling ability, can further enhance the model.

To address these challenges, multi-branch fusion architectures provide a new direction for gait recognition. By combining different types of neural networks, such as CNN and Bi-GRU, these models can fully exploit multi-dimensional features in gait data and enhance representational capability. Meanwhile, attention mechanisms, such as the SE module, help the model focus on critical information, further improving recognition performance. In addition, by replacing traditional fully connected layers with the novel KAN proposed by Liu et al. [27], a stronger non-linear modeling capacity is achieved.

This study introduces a multi-branch deep learning model (CNN-GRU-KAN) aimed at PD recognition and severity grading, designed explicitly for plantar pressure time-series data. Leveraging the same PhysioNet dataset, we contrast model performance between conventional machine learning algorithms and deep learning architectures on Parkinsonian gait classification. The model employs a one-dimensional CNN to capture local spatial and temporal characteristics from the input signals. Subsequently, a Bi-GRU, augmented with an attention mechanism, captures long-range dependencies and identifies critical gait segments within the temporal data. Following multi-level feature fusion, a KAN is introduced as an interpretable, spline-based function classifier head. This KAN layer enables flexible modeling of complex, non-linear decision boundaries for classification. Acknowledging the propensity of gait data for overfitting, the model systematically incorporates batch and layer normalization, as well as hierarchical dropout strategies, across its sub-modules. These techniques are implemented to enhance generalization capability and overall model stability. The primary contributions of this work are as follows.

- We designed an efficient multi-branch architecture for PD gait analysis that integrates a 1D-CNN branch with a two-layer Bi-GRU branch and fuses their outputs prior to a compact KAN head to address the limitations of purely CNN-based baselines in modeling long-range temporal dependencies, while effectively supporting both binary diagnosis and multi-class severity grading.
- We decoupled local spatial and long-range temporal modeling: the CNN (enhanced by SE attention) extracts localized spatial plantar pressure patterns, while the Bi-GRU (with temporal attention) captures bidirectional temporal context. A dedicated fusion MLP further provides dimensionality reduction and regularization, yielding a highly robust and data-efficient representation.
- We adopted KAN as the final classifier, replacing the linear head and removing the linear-weight constraint of conventional fully connected layers. By parameterizing with spline functions and optimizing end-to-end, KAN yields flexible decision boundaries.

The remainder of this article is organized as follows. Section 2 introduces the related work; Section 3 elaborates on the proposed method; Section 4 presents the experimental design and evaluation metrics; Section 5 showcases the experimental results and analysis; and Section 6 summarizes the entire article and outlines future work.

## 2. Related Work

In research on deep learning for PD detection, gait analysis is widely employed to enable the objective assessment of PD [28,29]. With their end-to-end feature learning capability, deep learning methods have gradually replaced traditional pipelines that rely on manual feature engineering. El Maachi et al. [17] introduced a deep learning method using a 1D-CNN, which uses parallel 1D convolutions to extract features from 18 channels of VGRF data, followed by feature fusion in fully connected layers for classification. Dong et al. [23] introduced a more complex static–dynamic temporal network. This architecture consists of two pathways: a static temporal pathway uses parallel 1D-ConvNets to process raw time-series independently, while a dynamic temporal pathway treats the shift of foot pressure points as “optical flow” and employs 16 parallel 2D-ConvNets to capture dynamic temporal features. In their study on wearable inertial sensors for freezing of gait (FOG) detection, Sigcha et al. [19] employed a single waist-mounted triaxial accelerometer and utilized parallel CNN and transformer structures for feature extraction. Borzi et al. [21] proposed a real-time FOG detection algorithm built on a multi-head CNN using single-IMU data. For visual data analysis, Zhao et al. [25] utilized smartphone-captured videos and human pose estimation to extract 3D joint coordinates for classification. Similarly, Pace et al. [30] introduced a markerless gait analysis approach using ViTPose to extract temporal, kinematic, and symmetric features from video sequences. Utilizing a support vector machine, they achieved an accuracy of 0.95 in PD detection and integrated SHAP for feature explainability, highlighting the clinical importance of knee angle and cadence.

With the increasing clinical demand for dynamic disease tracking, simple binary classification is no longer sufficient, making disease severity rating an important branch of research. To achieve fine-grained assessment, Vidya and Sasikumar [31] extracted gait features and combined them with a multi-class support vector machine (SVM) to successfully grade PD severity based on the H&Y scale. Following this trend, recent studies like Ji et al. [32] proposed a ST-CNN-Transformer fusion network that demonstrated excellent performance in both multimodal gait diagnosis and severity rating tasks. In the domain of ground reaction forces, Veeraragavan et al. [18] proposed an Artificial Neural Network (ANN) model utilizing solely VGRF data to diagnose PD with 97.4% accuracy and assess H&Y severity stages with 87.1% accuracy. Further exploring the potential of VGRF sensors, Navita et al. [33] designed a dual-stage machine learning model, applying a Random Forest Tree for PD classification and an Ensemble Regressor for severity assessment. Table 1 summarizes the representative objective PD detection algorithms in recent years.

Addressing the complexity of PD motor symptoms, researchers have also explored advanced spatio-temporal architectures and cross-modal techniques [36]. In gait analysis, Zhao et al. proposed ASTCapsNet [34] to jointly process multi-sensor data for high-level semantic features, and later introduced CorrMNN [35] for efficient multimodal gait signal fusion.

Despite the excellent diagnostic performance of recent multimodal or complex Transformer architectures, single-modality VGRF gait monitoring based on force plates retains an irreplaceable advantage in non-intrusive, home-based daily monitoring: it requires no active patient participation in specific writing or vocal tasks (i.e., passive monitoring) and incurs low deployment costs.

## 3. Methods

### 3.1. Method Overview

**Figure 1:**
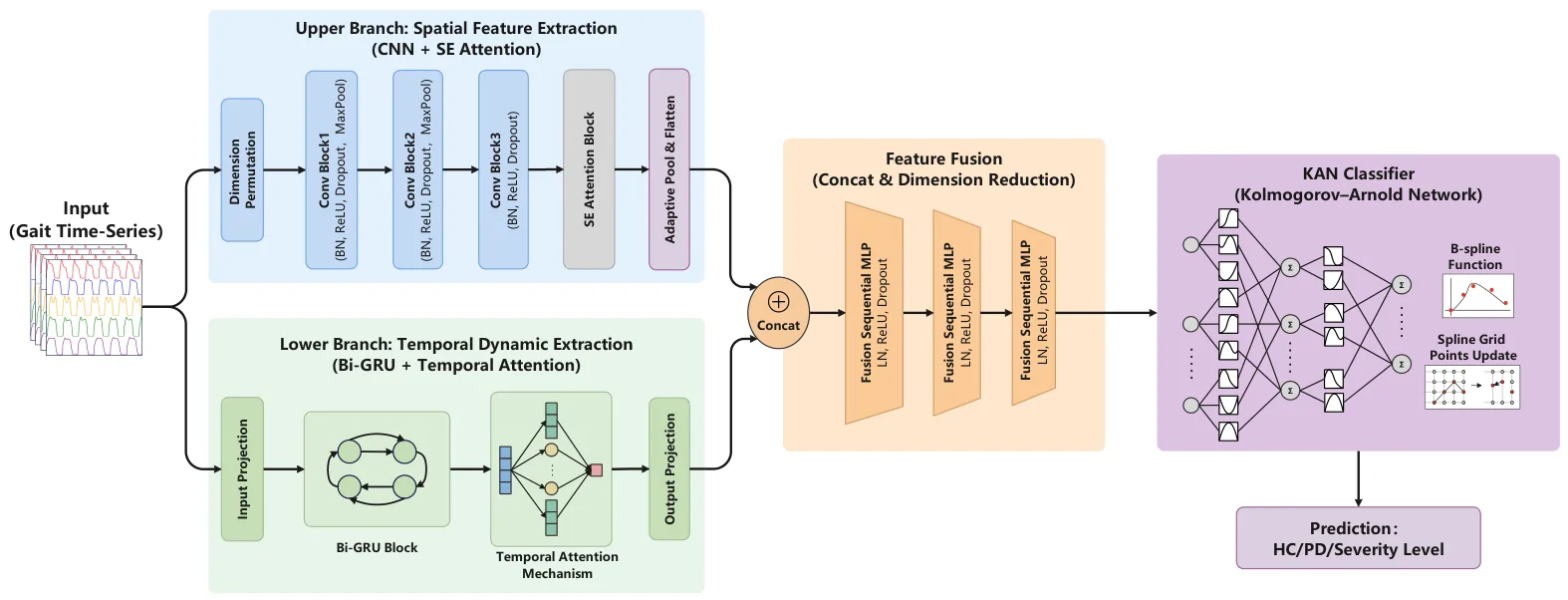
The architecture of the proposed CNN-GRU-KAN framework. The model adopts a dual-branch topology: an upper branch equipped with 1D-CNN and SE attention to extract local spatial pressure patterns, and a lower branch utilizing a Bi-GRU and temporal attention mechanism to capture long-range temporal dependencies. Following feature concatenation, the fused representations undergo dimension reduction via sequential MLP layers before being mapped by the KAN classification head to output the final predictions (healthy control/PD binary diagnosis/5-class severity level).

The proposed multi-branch deep learning framework is designed to capture spatial, temporal, and non-linear dynamic features from gait signals, thereby improving the accuracy and robustness of PD diagnosis and severity grading. As illustrated in Figure 1, the architecture consists of four primary components: an upper branch for spatial feature extraction (1D-CNN and SE attention), a lower branch for temporal dynamic extraction (Bi-GRU and temporal attention), a feature fusion and dimension reduction module, and a KAN classification head.

The design of this CNN-GRU-KAN architecture is fundamentally driven by the biomechanical characteristics of Parkinsonian gait. Since pathological gait anomalies manifest in both localized spatial pressure asymmetry and long-range rhythmic disruptions, we decoupled the feature extraction process. A 1D-CNN is employed specifically to capture short-term morphological variations in plantar pressure, while a parallel Bi-GRU tracks bidirectional arrhythmic temporal dependencies across the gait cycle. Finally, we integrated a KAN classification head to map these fused spatio-temporal representations to binary diagnostic outcomes and discrete UPDRS severity scales. This classification involves highly complex decision boundaries where traditional MLPs with fixed activations are prone to overfitting. Consequently, the learnable B-spline functions of KAN provide the adaptive mathematical flexibility required to accurately delineate these intricate clinical manifolds.

### 3.2. Spatial Feature Extraction Branch

In the proposed architecture, the upper branch is specifically optimized to extract local spatial representations and short-term pressure variations from the gait signals. This branch consists of three cascaded 1D convolutional layers designed to process the 18-channel VGRF input. As the data propagates through these layers, the number of feature channels is progressively expanded from 18 to 32, 64, and finally 96. To enhance model stability and mitigate the risk of overfitting during training, each convolutional layer is followed by batch normalization (BatchNorm1d) and dropout regularization. Additionally, maxPool1d layers are interspersed between convolutions to perform downsampling and feature length compression, effectively broadening the receptive field of the filters.

1D-CNNs are particularly effective for modeling localized patterns in time-series data without requiring manual feature engineering [25]. In our model, the 1D convolution operation slides learnable filters across the input sequence to perform a weighted summation over local receptive fields. This process allows the network to automatically capture discriminative pathological markers, such as abnormal force distributions during specific phases of the gait cycle. Formally, given an input sequence *x* and a convolution kernel *w* of length *K*, the output feature y[i] at position *i* is computed as:

(1) $$y\left[i\right]=\sum_{k=0}^{K-1}x\left[i+k\right]\cdot w\left[k\right]+b$$

where y[i] denotes the output feature at index *i*, x[i+k] represents the input sample offset by *k*, w[k] is the *k*-th kernel coefficient, *K* is the kernel length, and *b* is a learnable bias term that shifts the activation response.

Following the final convolutional layer, an SE attention module is integrated. This mechanism dynamically recalibrates channel-wise feature weights, enabling the branch to explicitly emphasize critical spatial sensor channels while suppressing less relevant noise. Ultimately, this branch provides a refined, high-dimensional spatial representation of the foot pressure dynamics for subsequent feature fusion.

### 3.3. Temporal Dynamic Extraction Branch

While the CNN branch extracts local spatial features from the 18 VGRF channels, Parkinsonian gait abnormalities also involve continuous temporal changes, including freezing of gait and rhythm variations. To capture these long-range temporal dependencies within the 100-step gait windows, we employ a parallel temporal branch. The core temporal modeling relies on the gated recurrent unit (GRU) [37].

**Figure 2:**
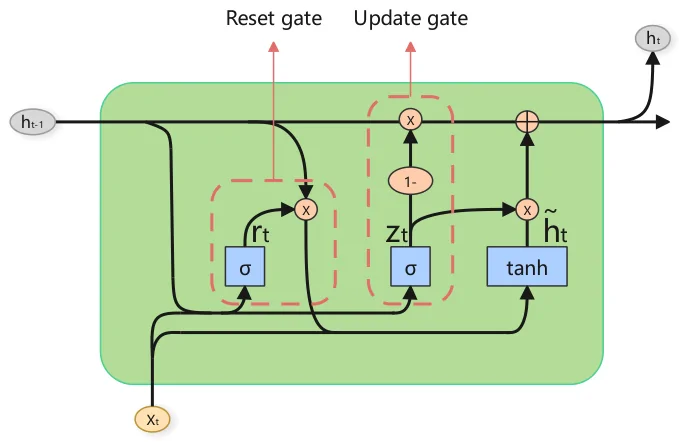
Gated recurrent unit.

The standard GRU elegantly mitigates the vanishing gradient problem found in traditional RNNs by regulating information flow through gating mechanisms. As shown in Figure 2, at any given time step *t*, the update gate $z_{t}$ and the reset gate $r_{t}$ control the retention of previous states and the incorporation of new inputs. The internal state transitions are formulated as follows:

(2) $$\begin{array}{cc} z_{t} & =\sigma \;\left(W_{z}x_{t}+U_{z}h_{t-1}+b_{z}\right) \end{array}$$

(3) $$\begin{array}{cc} r_{t} & =\sigma \;\left(W_{r}x_{t}+U_{r}h_{t-1}+b_{r}\right) \end{array}$$

(4) $$\begin{array}{cc} {\tilde{h}}_{t} & =\tanh\;\left(W_{h}x_{t}+U_{h}\left(r_{t}⊙h_{t-1}\right)+b_{h}\right) \end{array}$$

(5) $$\begin{array}{cc} h_{t} & =\left(1-z_{t}\right)⊙h_{t-1}+z_{t}⊙{\tilde{h}}_{t} \end{array}$$

where $x_{t}$ is the input vector, $h_{t}$ and ${\tilde{h}}_{t}$ denote the current and candidate hidden states, respectively, and $h_{t-1}$ is the hidden state from the previous time step. The matrices W and U represent the learnable weights, b are the bias terms, σ(·) is the sigmoid activation function, and ⊙ denotes element-wise multiplication.

While a standard unidirectional GRU processes sequences chronologically, it lacks access to future contextual information. To construct a comprehensive temporal representation, we upgrade the core unit to a bidirectional GRU (Bi-GRU). The Bi-GRU processes the sequence simultaneously in both forward (${\vec{h}}_{t}$) and backward (${\overset{\leftarrow }{h}}_{t}$) directions:

(6) $$\begin{array}{cc} {\vec{h}}_{t} & =\mathrm{GRU}(x_{t},{\vec{h}}_{t-1}) \end{array}$$

(7) $$\begin{array}{cc} {\overset{\leftarrow }{h}}_{t} & =\mathrm{GRU}(x_{t},{\overset{\leftarrow }{h}}_{t+1}) \end{array}$$

The bidirectional representation for time step *t* is formed by concatenating the states from both directions:

(8) $$h_{t}^{\mathrm{bi}}=\left[{\vec{h}}_{t}\;;\;{\overset{\leftarrow }{h}}_{t}\right]$$

Finally, rather than treating all 100 time steps equally, a temporal attention mechanism is applied to the sequence of hidden states $h_{t}^{\mathrm{bi}}$. This allows the network to dynamically assign higher weights to critical phases of the gait cycle (e.g., heel strike or toe-off) that contain more discriminative pathological features, ultimately outputting a context-aware, robust temporal representation for subsequent fusion.

### 3.4. Feature Fusion

To integrate the complementary information from the spatial and temporal branches, we employ a feature fusion mechanism. The flattened outputs from the CNN branch (spatial features, dimension $D_{s}$) and the Bi-GRU branch (temporal features, dimension $D_{t}$) are concatenated along the feature dimension to form a unified representation:

(9) $$F_{\mathrm{concat}}=F_{\mathrm{spatial}}⊕F_{\mathrm{temporal}}$$

where ⊕ denotes the concatenation operation, yielding $F_{\mathrm{concat}}$ with a dimension of $D_{s}+D_{t}$.

Directly feeding this high-dimensional concatenated vector into the subsequent classifier could lead to parameter explosion and severe overfitting. Therefore, $F_{\mathrm{concat}}$ is passed through a dedicated feature fusion module for dimensionality reduction and feature interaction. This module consists of sequential multi-layer perceptron (MLP) blocks, where each block is equipped with layer normalization (LN), ReLU activation, and dropout regularization. This step effectively projects the heterogeneous features into a compact, joint spatio-temporal representation vector $x_{0}$, ensuring the model leverages both local spatial patterns and long-range sequential dependencies without sacrificing generalization.

### 3.5. Kolmogorov–Arnold Network

In conventional deep learning models, classification relies on fully connected layers, which are simple but prone to overfitting owing to the high parameter count. This study introduces a Kolmogorov–Arnold network (KAN) as the final classification layer. Developed by Liu et al. [27], based on the Kolmogorov–Arnold representation theorem, KAN differs from traditional neural networks by using learnable univariate function combinations parameterized by B-spline basis functions, instead of predetermined activation functions and linear weights.

**Figure 3:**
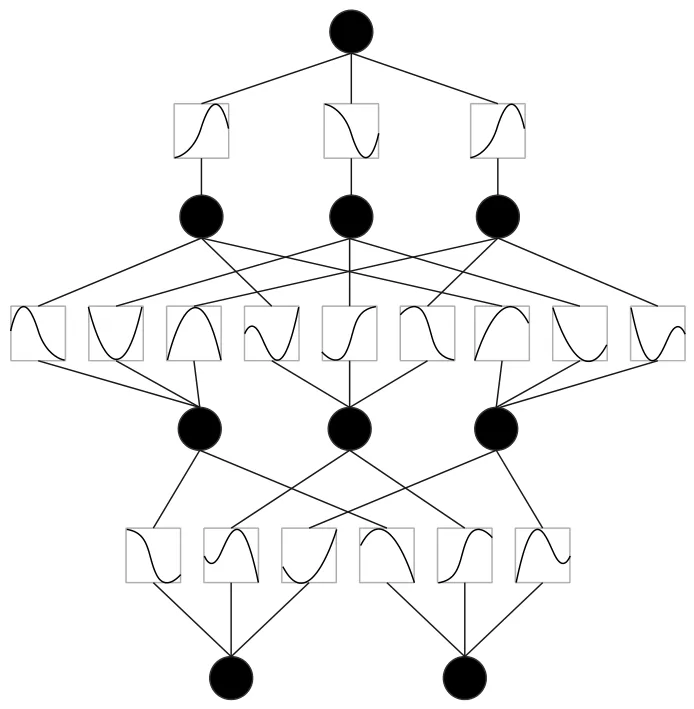
Kolmogorov–Arnold networks.

As shown in Figure 3, KAN’s mechanism is essentially different from traditional networks. Unlike conventional neural networks that apply static activations at neurons, KAN replaces the weight sections with trainable activation functions that vary for each section. For a KAN with n-dimensional input and output, it is defined as a one-dimensional function matrix. Specifically, a KAN layer can be represented as follows:

(10) $$x_{l+1,j}=\sum_{i=1}^{n_{l}}{\tilde{x}}_{l,j,i}=\sum_{i=1}^{n_{l}}\phi_{l,j,i}\left(x_{l,i}\right),\;j=1,\ldots ,n_{l+1}$$

The matrix form is given by:

(11) $$x_{l+1}=\left(\begin{array}{ccc} \phi_{l,1,1}\left(\cdot \right) & \ldots & \phi_{l,1,n_{l}}\left(\cdot \right)\\ \phi_{l,2,1}\left(\cdot \right) & \ldots & \phi_{l,2,n_{l}}\left(\cdot \right)\\ \vdots & \ddots & \vdots \\ \phi_{l,n_{l+1},1}\left(\cdot \right) & \ldots & \phi_{l,n_{l+1},n_{l}}\left(\cdot \right) \end{array}\right)x_{l}$$

Among them, (l,i) denotes the *i*-th neuron in layer *l*, and $x_{l,i}$ is its activation. For the basis function $\phi_{l,j,i}$, the pre-activation is $x_{l,i}$, and the post-activation is ${\tilde{x}}_{l,j,i}\equiv \phi_{l,j,i}\left(x_{l,i}\right)$.

In general, a KAN is formed by multiple KAN layers. When provided with an input vector $x_{0}$, it generates the corresponding output:

(12) $$\mathrm{KAN}\left(x_{0}\right)=\left(\Phi_{L-1}\circ \Phi_{L-2}\circ \ldots \circ \Phi_{1}\circ \Phi_{0}\right)x_{0}$$

Among them, the intermediate matrix $\Phi_{l}$ is the function matrix corresponding to the *l*-th KAN layer.

## 4. Experiments

### 4.1. Database

The proposed framework was evaluated using three independent Parkinsonian gait sub-cohorts derived from the PhysioNet database. These cohorts are designated as Ga (natural overground walking), Ju (dual-task cognitive walking), and Si (controlled treadmill walking) [38,39,40]. They represent three distinct clinical testing paradigms and provide a comprehensive spectrum of motor assessments. Regarding the testing conditions, subjects in the Ga and Ju cohorts were instructed to walk at their usual, self-selected comfortable pace on level ground. In contrast, the Si cohort performed walking tasks on a motorized treadmill at strictly controlled speeds. The primary diagnostic modality comprises 18-channel VGRF signals continuously recorded during locomotion.

To capture localized dynamic temporal patterns and facilitate deep feature extraction, a sliding-window technique was systematically applied to segment the continuous VGRF time series data. The window size was strictly defined as 100 frames. This corresponds to approximately one second of a physiological gait cycle. A sliding stride of 50 frames was used to ensure temporal continuity while adequately augmenting the training distribution.

The dataset supports both binary classification tasks (PD versus non-PD) and multi-class severity staging (e.g., based on H&Y or UPDRS stages). Demographic statistics of the subjects are presented in Table 2, and the stratification by UPDRS rating is reported in Table 3.

**Figure 4:**
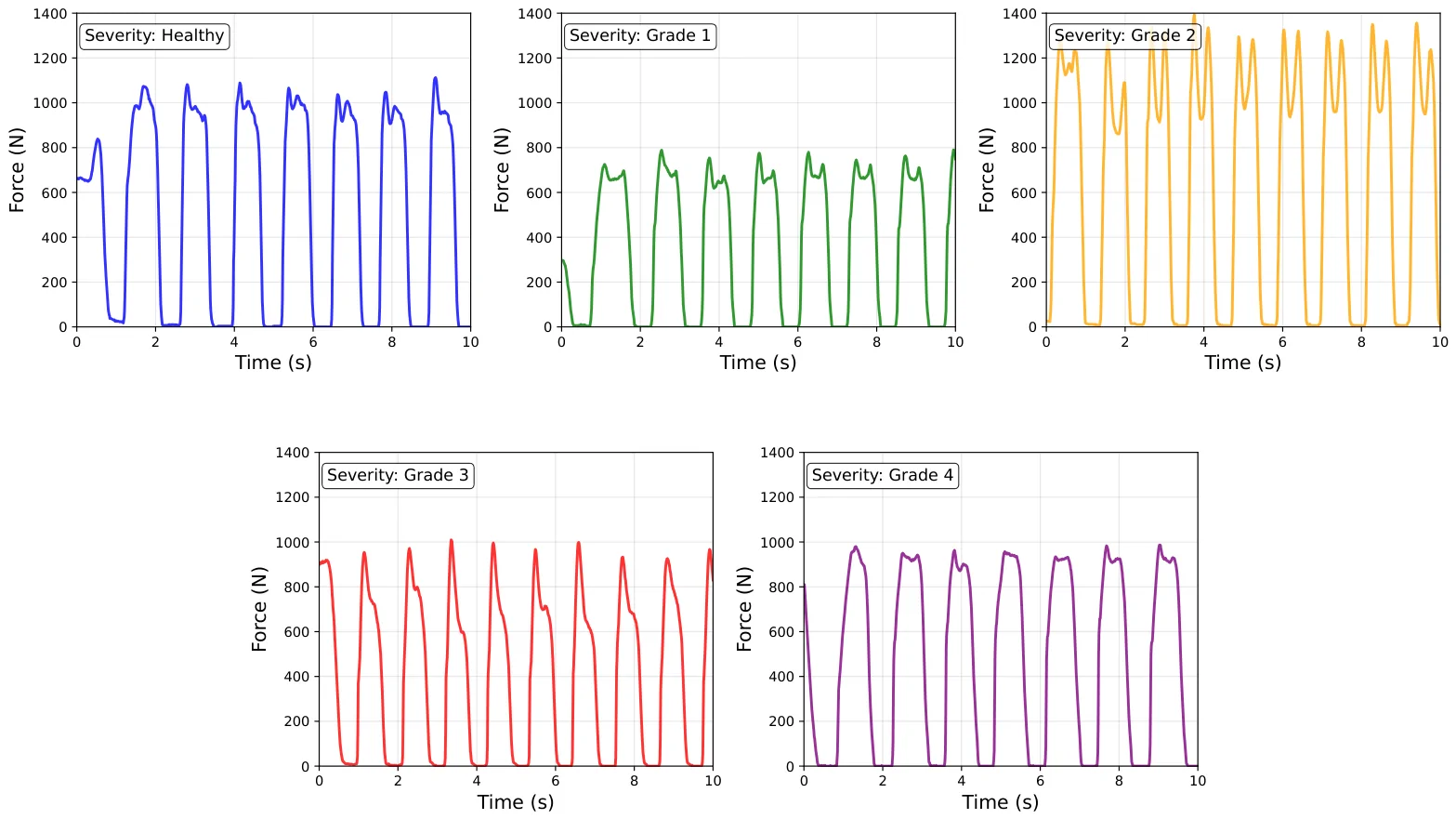
Comparative analysis of left-foot VGRF waveforms across PD severity stages. (Note: These waveform plots were generated by the authors using representative raw time-series data extracted from the publicly available PhysioNet databases).

Figure 4 illustrates a comparative analysis of total left-foot VGRF waveforms between HC and PD patients. Control subjects exhibit consistent periodic patterns with stable peak forces. Progressive disease severity correlates with distinct waveform alterations: increased peak force variability, fluctuations during the mid-stance phase, and irregular periodicity. These biomechanical deviations align with characteristic motor control deficits and gait abnormalities in PD, serving as an objective biomarker for disease severity assessment. Compared to controls, PD patients demonstrate greater gait asymmetry in VGRF signals, with significantly reduced force exertion in the left foot. Such deviations may substantially impair ambulatory function and mobility, potentially necessitating more intensive therapeutic interventions and clinical management strategies.

### 4.2. Parkinson’s Severity Prediction

Beyond binary PD detection, this study extends the proposed framework to quantify disease severity using gait data. The MDS-UPDRS total score was utilized as the primary evaluation criterion, owing to its clinical prevalence in standardized PD assessment [9]. To convert continuous UPDRS scores into a discrete severity grading framework, the score range was stratified into five levels based on clinical relevance and data distribution:

- Grade 0: UPDRS<5;
- Grade 1: 5≤UPDRS<15;
- Grade 2: 15≤UPDRS<25;
- Grade 3: 25≤UPDRS<35;
- Grade 4: UPDRS≥35.

The multi-branch architecture remains consistent with the binary detection task, with the exception of the classification head. The output dimension of the KAN classifier was adjusted from 2 to 5 to accommodate the designated severity grades. Training followed the identical optimization and regularization protocol established for binary diagnosis. This gait-based severity prediction approach provides an objective, noninvasive auxiliary tool for clinical assessment, assisting clinicians in monitoring disease progression and evaluating treatment efficacy.

### 4.3. Evaluation Metrics

To rigorously evaluate the effectiveness of the proposed approach for gait-based PD identification and severity classification, we employed multiple standard metrics. Here, TP counts positive instances correctly classified as positive; TN counts negative instances correctly classified as negative; FP counts negative instances incorrectly labeled as positive; FN counts positive instances incorrectly labeled as negative. The evaluated metrics are calculated as follows:

- Sensitivity (Se%):
  (13) $$\mathrm{Sensitivity}=\frac{\mathrm{TP}}{\mathrm{TP}+\mathrm{FN}}$$
- Specificity (Sp%): Reflects the model’s capability to correctly identify true negative samples (e.g., healthy controls).
  (14) $$\mathrm{Specificity}=\frac{\mathrm{TN}}{\mathrm{TN}+\mathrm{FP}}$$
- Accuracy (Acc%): Represents the overall proportion of correctly classified instances among all predictions.
  (15) $$\mathrm{Accuracy}=\frac{\mathrm{TP}+\mathrm{TN}}{\mathrm{TP}+\mathrm{TN}+\mathrm{FP}+\mathrm{FN}}$$

### 4.4. Evaluation Strategies

To comprehensively validate the model’s robustness, diagnostic stability, and cross-cohort generalization, we established a multi-tiered evaluation protocol. A paramount principle stringently enforced across all experimental configurations was strict subject-level isolation. This is critical for eradicating data leakage and ensuring the clinical validity of the predictions.

#### 4.4.1. Integrated Dataset Evaluation

**Figure 5:**
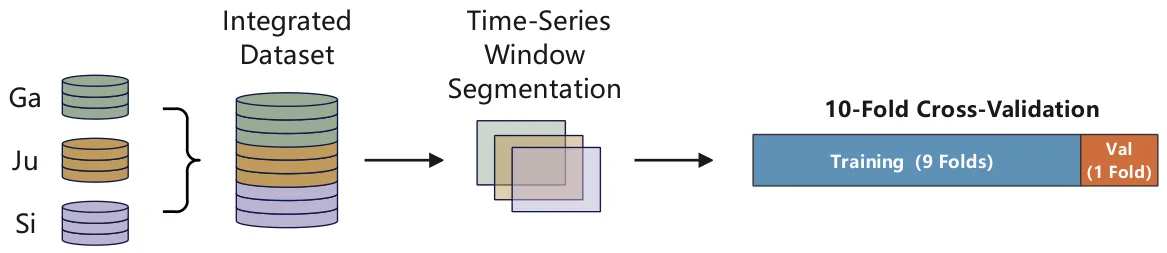
Integrated dataset splitting. Val: validation subset.

As depicted in Figure 5, the three distinct cohorts (Ga, Ju, Si) are combined into a single dataset. We deploy a stratified 10-fold cross-validation scheme partitioned explicitly by unique patient identifiers (Patient IDs). This isolation guarantees that overlapping gait sequences from a single subject never concurrently exist in the training and validation sets. Furthermore, each fold maintains a consistent distribution ratio of 70% PD patients to 30% healthy controls. This mitigates evaluation bias stemming from class imbalance. The final performance metrics are computed by pooling the predictive outputs across all 10 validation folds. This approach ensures a robust and unbiased representation of diagnostic capability.

#### 4.4.2. Single Dataset Evaluation

**Figure 6:**
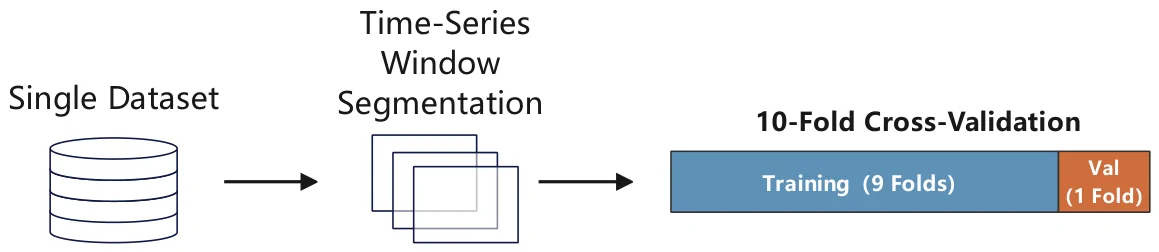
Single dataset splitting. Val: validation subset.

To assess the feature extraction stability of the proposed architecture under specific locomotor tasks, we independently executed the subject-level 10-fold cross-validation within each individual sub-cohort (Ga, Ju, Si), as illustrated in Figure 6. This intra-dataset evaluation delineates the model’s performance fidelity when confronted with isolated and task-specific clinical scenarios. Examples include pure treadmill ambulation versus dual-task cognitive interference.

#### 4.4.3. Cross-Dataset Evaluation

**Figure 7:**
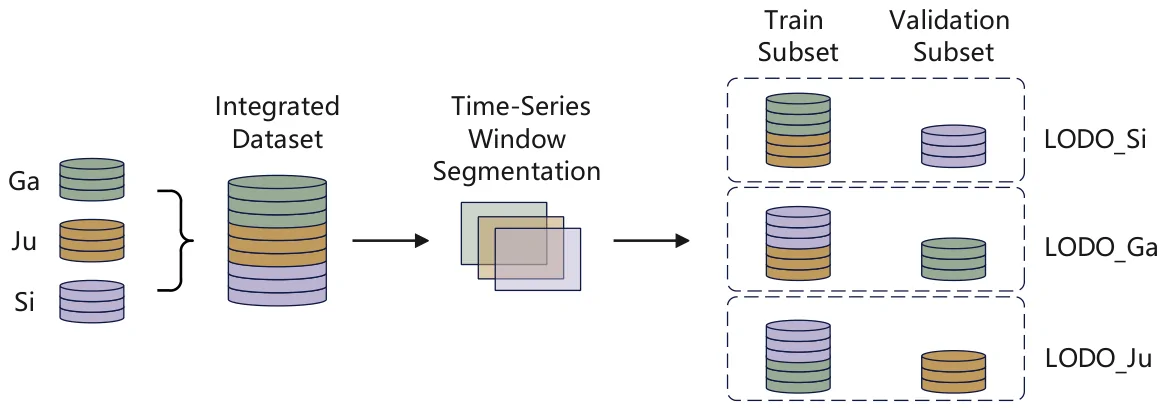
Cross-dataset splitting. Val: validation subset.

We introduced a leave-one-dataset-out (LODO) evaluation framework to rigorously test the model’s generalization capacity under extreme domain shifts and environmental confounding variables (e.g., treadmill versus natural overground walking). This simulated real-world clinical deployments where the acquisition environment is fundamentally unseen. In this configuration, as detailed in Figure 7, three distinct testing scenarios are constructed. Each scenario iteratively isolates one entire sub-cohort (Ga, Ju, or Si) as the external testing set, while the remaining two are concatenated for model training.

The training process for each LODO scenario was independently repeated 10 times under identical data partitioning conditions. This repeated training negated statistical anomalies arising from the stochastic initialization of deep learning parameters. We report the mean metrics and corresponding standard deviations across these independent runs to ensure absolute statistical reliability.

### 4.5. Ablation Study Design

To rigorously validate the effectiveness and necessity of each component within the proposed CNN-GRU-KAN architecture, we constructed three degraded baseline variants for comprehensive ablation testing. These variants were evaluated under identical experimental settings:

- Only-GRU (No CNN branch):Removing the 1D-CNN and SE attention module, relying solely on the Bi-GRU branch for pure temporal feature extraction.
- Only-CNN (No Bi-GRU branch): Removing the Bi-GRU and temporal attention module, relying solely on the CNN branch for spatial feature extraction.
- No KAN (Standard MLP): Retaining the dual-branch spatio-temporal fusion structure, but replacing the novel KAN classification head with conventional fully connected MLP layers.

### 4.6. Implementation Details

The proposed CNN-GRU-KAN spatial-temporal framework was systematically decoupled into distinct functional branches. Each branch was parameterized to optimize feature representation while mitigating the risk of overfitting.

- 1D-CNN branch: Localized spatial plantar pressure patterns are extracted using a three-layer one-dimensional convolutional cascade. The channel dimensions were progressively expanded (18→32→64→96). Kernel sizes of 7, 5, and 3 were utilized, respectively. Downsampling was achieved by applying a stride of 2 in the initial two convolutional layers. To stabilize the internal covariate shift, each convolutional operation was sequentially followed by a BatchNorm1d layer, a ReLU activation function, and a dropout layer (p=0.5). The branch terminates with an SE attention module (reduction ratio = 16) for channel-wise feature recalibration. This is followed by an AdaptiveAvgPool1d layer, yielding a compact 96-dimensional spatial representation.
- Bi-GRU branch: Long-range bidirectional temporal dependencies were modeled via a 2-layer Bi-GRU. The hidden state dimension was configured to 64, generating a concatenated output dimension of 128. Subsequently, a temporal attention mechanism utilizing a tanh activation function was applied. This dynamically assigns attention weights to critical phases of the gait cycle, yielding a robust context-aware temporal feature vector.
- Feature fusion and dimensionality reduction: The 96-dimensional spatial and 128-dimensional temporal vectors were concatenated to form a unified 224-dimensional joint representation. To execute cross-domain feature interaction and dimensionality reduction, this vector propagates through a 3-layer sequential MLP. The MLP layers progressively project the feature space through dimensions of 128, 64, and finally 32. Each layer integrates LN, ReLU activation, and dropout regularization to prevent parameter explosion.
- Kolmogorov–Arnold network classifier head: Replacing conventional linear fully connected layers, the architecture employs a KAN classification head to model highly non-linear decision boundaries. The hidden nodes are sequentially configured as $\left[32,16,N_{classes}\right]$. The learnable univariate functions are parameterized by second-order B-splines (Spline Order = 2) distributed across a grid size of 3. The SiLU function is employed as the baseline activation.
- Training hyperparameters: The network was optimized end-to-end using the AdamW optimizer. The base learning rate was $5\times 10^{-4}$ and the weight decay coefficient was $1\times 10^{-2}$. The maximum batch size was set to 64, and the training strictly converged over 100 fixed epochs. Focal Loss was employed as the primary objective function to counteract class imbalance during optimization.

## 5. Results and Discussion

### 5.1. Models Used for Comparison

To thoroughly evaluate the performance of our proposed CNN-GRU-KAN hybrid architecture, we selected several representative advanced methods for comparative analysis. These baselines were explicitly chosen to represent the evolutionary trajectory of gait analysis algorithms: from traditional machine learning approaches (e.g., MLP, SVM, RF) relying on basic features, to advanced deep learning architectures (e.g., ANN, Static-Dynamic Net) designed for complex spatio-temporal data modeling. The performance comparison of these methods is summarized in Table 4.

**Figure 8:**
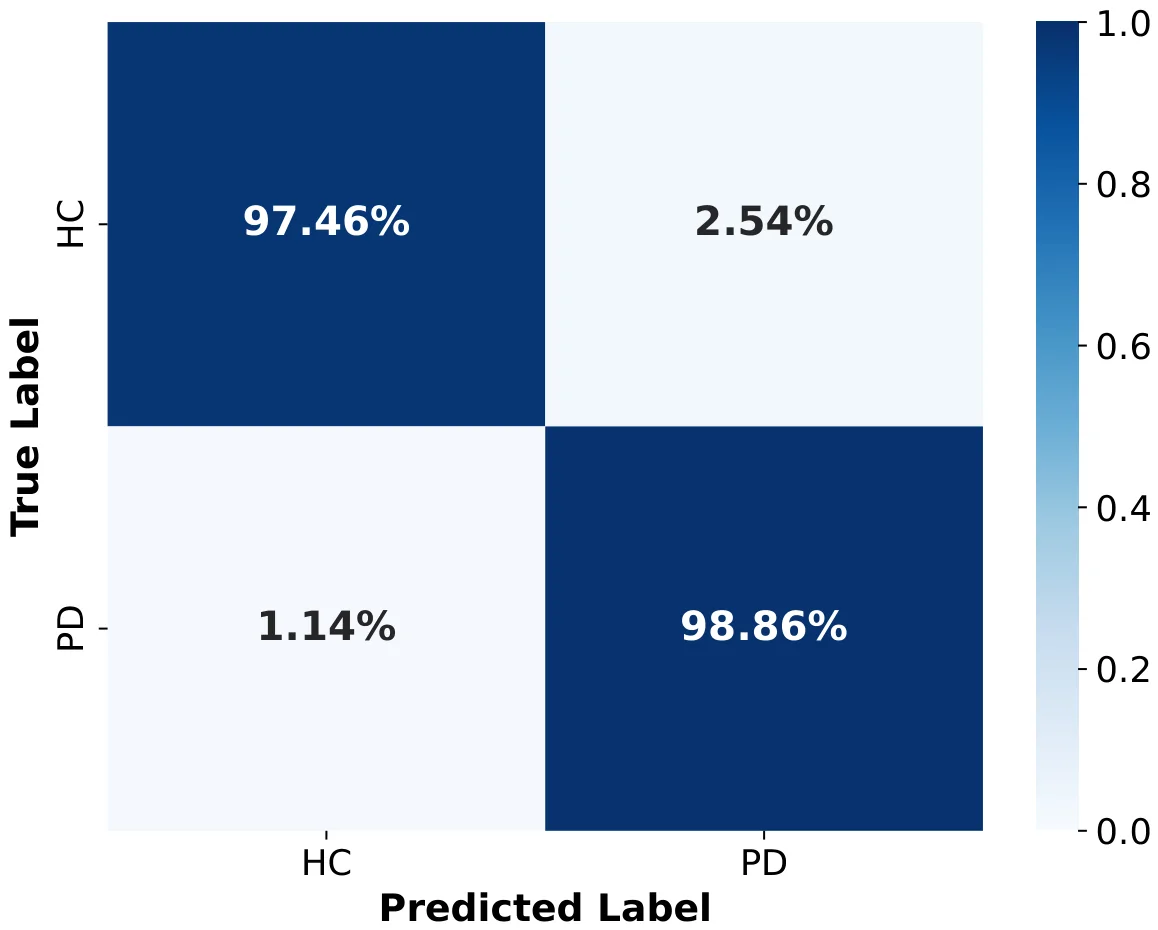
Normalized confusion matrix for PD classification.

The confusion matrix in Figure 8 illustrates the model’s classification performance in binary PD detection, detailing the normalized distribution of predicted versus actual labels for HC and PD patients. This distribution explicitly demonstrates the model’s robust diagnostic capability.

### 5.2. Ablation Study Results

Table 5 summarizes the performance of these variants. Removing the temporal branch (Only-CNN) or the spatial branch (Only-GRU) leads to severe performance degradations. Overall accuracy plummets to 91.77% and 90.84%, respectively. This steep decline demonstrates that relying solely on spatial or temporal features is insufficient for robust PD detection. Notably, the sensitivity drops to 88.26% in the Only-GRU configuration.

Furthermore, substituting the proposed KAN classifier with a conventional fully connected MLP head (No-KAN variant) results in an accuracy drop from 98.43% to 97.15%. This measurable performance gap validates our hypothesis. The dynamic, spline-based non-linear transformations of the KAN module provide superior modeling capabilities. They effectively delineate the complex, high-dimensional decision boundaries between pathological and healthy gait dynamics.

### 5.3. Results of Single Dataset Evaluation

To assess the model’s feature extraction fidelity across distinct locomotor tasks, we evaluated the architecture independently within each sub-cohort (Ga, Ju, Si). This process strictly adhered to the 10-fold subject-isolated paradigm. The results presented in Table 6 elucidate the nuances of task-specific performance.

The model achieves optimal performance on the Si dataset (treadmill walking) with an accuracy of 97.87% and a sensitivity of 98.83%. This is likely due to the highly controlled nature of motorized treadmills, which yield consistent rhythmic patterns. Conversely, performance slightly degrades on the Ga dataset (natural overground walking), yielding an accuracy of 94.70%. This inherently reflects the increased biomechanical variability and unconstrained kinematic trajectories associated with natural ambulation. Nevertheless, the model maintains high sensitivity and specificity across all three isolated environments. This establishes a solid baseline for task-agnostic PD detection.

### 5.4. Results of Cross-Dataset Evaluation

A fundamental bottleneck impeding the clinical deployment of deep learning models in gait analysis is the susceptibility to domain shifts. To critically stress-test our model against such shifts, we executed the LODO generalization protocol.

As summarized in Table 7, the proposed CNN-GRU-KAN architecture exhibits strong cross-cohort robustness. The model consistently sustains high performance levels when completely unseen cohorts were utilized as external validation benchmarks. Specifically, the model maintains an accuracy of 97.93% and a sensitivity of 98.33% when tested on entirely unseen natural walking data (LODO_Ga). Similar generalization capacities are observed in LODO_Ju (98.13% ACC) and LODO_Si (98.34% ACC). These marginal performance variances prove that the multi-branch architecture extracts invariant, generalizable pathological biomarkers of Parkinsonian gait. It successfully avoids overfitting to cohort-specific acquisition artifacts or environmental confounds.

Specifically, acquisition artifacts (e.g., irregular start/stop steps or transient sensor noise) were strictly controlled prior to training through the overlapping sliding-window segmentation, which effectively isolates steady-state gait cycles. Meanwhile, environmental confounding variables (such as the biomechanical differences between motorized treadmills and natural overground surfaces) were intrinsically mitigated by the model’s decoupled spatial-temporal feature extraction, as validated by this LODO protocol. Consequently, the model successfully avoided overfitting to cohort-specific conditions.

### 5.5. Parkinson Severity Prediction

Beyond binary detection, evaluating the model’s utility for continuous clinical disease tracking is paramount. Table 8 compares the severity staging performance of our five-class KAN-based framework with existing approaches. Our method achieves a state-of-the-art accuracy of 93.46%, outperforming both traditional SVM-RBF models and the advanced Static-Dynamic Net.

**Figure 9:**
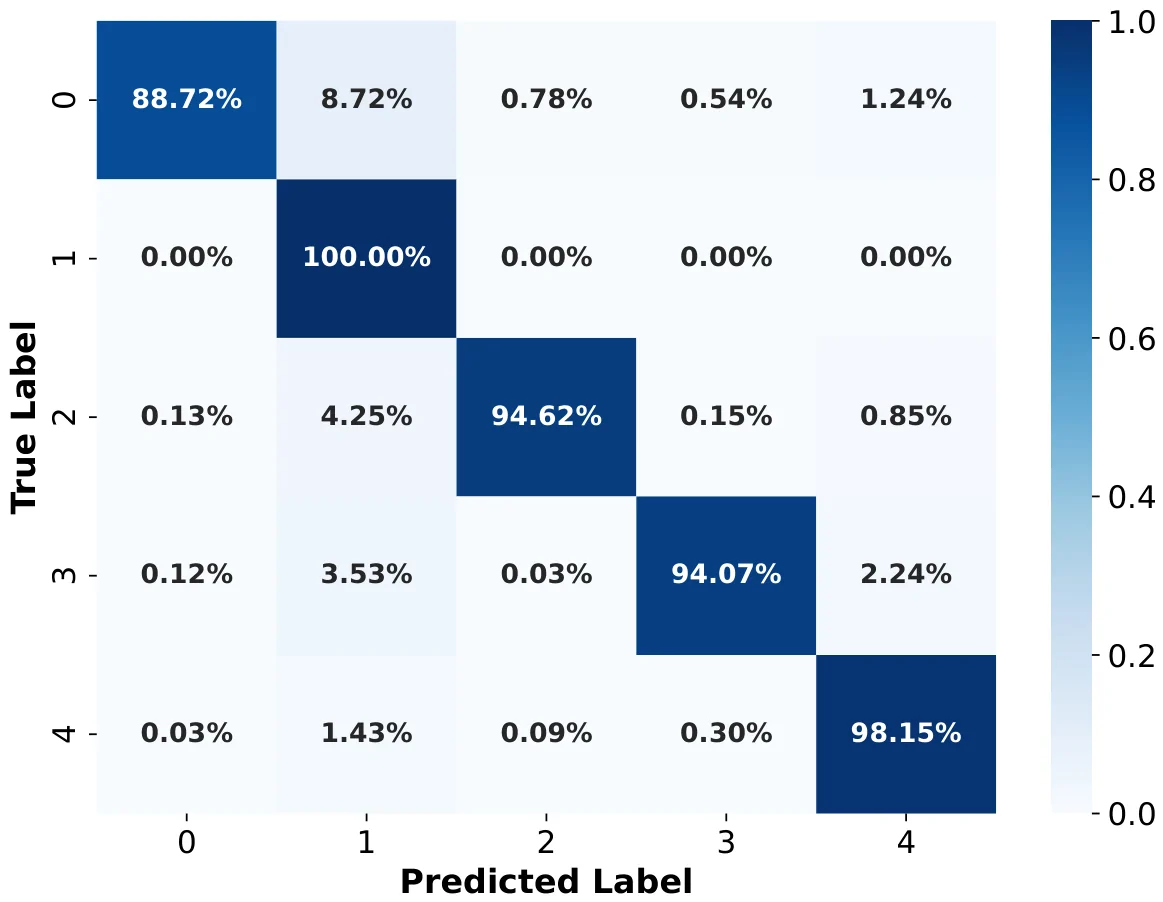
Normalized confusion matrix of the UPDRS classification.

As depicted in Figure 9, the corresponding normalized confusion matrix exhibits a highly concentrated diagonal distribution. Crucially, most off-diagonal misclassifications occur strictly between adjacent severity levels (e.g., confusing Grade 0 with Grade 1). The model successfully avoids severe diagnostic errors, such as misclassifying healthy controls as severe PD cases.

### 5.6. Discussion

The extensive experimental validation highlights several core strengths of the proposed CNN-GRU-KAN architecture. Clinically, the model’s ability to maintain high generalization across unseen domains (as evidenced by the LODO evaluation) indicates that it successfully extracts intrinsic pathological biomarkers of Parkinsonian gait, rather than overfitting to specific environmental confounds. Methodologically, decoupling spatial and temporal extraction allows the model to interpret localized asymmetric foot pressure distributions (via CNN) simultaneously with long-term arrhythmic freezing of gait patterns (via Bi-GRU).

Despite these promising results, this study acknowledges certain limitations that warrant further investigation. First, the current data preprocessing pipeline does not manually normalize the VGRF signals by the subjects’ body weight. Although our internal normalization layers computationally handle signal scaling, explicit biomechanical weight normalization could potentially isolate pathological force variations more effectively. Second, while the physical interpretation of VGRF asymmetry is intuitive, the specific non-linear decision boundaries generated by the KAN module remain mathematically opaque, requiring further explainable AI (XAI) integration. Finally, the overall demographic diversity of the dataset is relatively limited, and the effectiveness of the model in real, unconstrained clinical settings needs further verification.

## 6. Conclusions

Early diagnosis and accurate assessment of PD remain significant challenges in the field of neurology. Traditional diagnostic methods lack standardized and quantifiable evaluation measures. This study proposes a novel multi-branch fused deep learning model for intelligent diagnosis and severity assessment of PD based on continuous pressure sensor data. Under rigorous evaluation protocols, including strict subject-level isolation and cross-cohort testing, the proposed model achieved 98.43% accuracy for Parkinson’s gait recognition and 93.46% for the multi-class severity prediction task.

Furthermore, the proposed method holds considerable clinical value. It provides a noninvasive and objective means of PD diagnosis, mitigating the subjectivity and inter-rater variability of traditional observational approaches. The model is capable of simultaneous disease diagnosis and severity assessment, offering comprehensive quantitative metrics to support clinical decision-making. The pressure sensor-based data acquisition is unobtrusive and practical, facilitating potential deployment in community and home environments. With the growing adoption of wearable smart sensors, this method serves as a highly generalizable technical pathway for early screening and continuous remote monitoring of PD.

For future work, we plan to explore the following directions: First, collect larger-scale, multi-center clinical data, including patients of different ages, genders, and disease stages, to validate the model’s long-term generalization capability. Second, explicitly incorporate physiological priors (such as body-weight normalization) into the data-processing pipeline to enhance biomechanical fidelity. Third, integrate other biomarkers (such as voice and upper-limb tremor) to construct a comprehensive multimodal diagnostic system. With ongoing progress in AI and biomedical sensors, the proposed approach is poised to support early diagnosis, individualized treatment, and continuous surveillance of PD, thereby contributing to better patient quality of life.

## Figures and Tables

**Table 1:** Summary and comparison of representative objective PD detection algorithms.

Study/Method	Modality	Task	Advantages
1D-Convnet [17]	Gait	Binary/Severity	Efficient extraction of spatial local features for baseline diagnosis.
Static-Dynamic Net [23]	Gait	Binary/Severity	Explores dynamic “optical flow” features through parallel pathways.
MCSVM [31]	Gait	Severity	Spatio-temporal features are extracted through kinematic analysis, combined with correlation-based feature selection and multiple regression normalization.
ASTCapsNet [34]	Gait	Severity	Captures high-level semantic correlations via complex capsule routing.
CorrMNN [35]	Gait	Severity	Achieves high accuracy when strictly aligned multimodal data is available.
ANN [18]	Gait	Binary/Severity	Achieves high accuracy for both diagnosis and H&Y severity assessment using solely VGRF data.
RFT-ER [33]	Gait	Binary/Severity	Employs a dual-stage machine learning model with recursive feature elimination for highly accurate severity regression.
RFE-SVM [30]	Gait	Binary	Utilizes markerless pose estimation (ViTPose) and integrates SHAP for clinical feature explainability.
ST-CNN-Transformer [32]	Gait	Binary/Severity	Integrates statistical tests and Transformers for comprehensive staging.

**Table 2:** Statistics of control group and PD patients.

Dataset	Group	Total Subjects	Male	Female	Height (m)	Weight (kg)
Ju [40]	Healthy	26	12	14	1.83	66.8
	PD patient	29	16	13	1.87	75.1
Ga [39]	Healthy	18	10	8	1.68	74.2
	PD patient	29	20	9	1.67	73.1
Si [38]	Healthy	29	18	11	1.69	71.5
	PD patient	35	22	13	1.66	70.3

**Table 3:** Distribution of subjects across UPDRS categories.

Dataset	Grade 0	Grade 1	Grade 2	Grade 3	Grade 4
Ju [40]	26	2	15	8	4
Ga [39]	18	0	8	11	10
Si [38]	29	0	5	11	17

**Table 4:** Performance comparison of PD detection methods.

Methods	Sensitivity (%)	Specificity (%)	Accuracy (%)
MLP [26]	88.9	82.2	88.9
NB [26]	n/a	n/a	76.1
RF [26]	n/a	n/a	86.9
SVM [41]	n/a	n/a	84.5
GLRA [41]	n/a	n/a	82.8
ANN [18]	n/a	97.1	97.4
RFT [33]	97	95	97.5
RFE-SVM [30]	90	97	95
Static-Dynamic Net [23]	98.1	93.3	96.7
Proposed Method	98.86	97.46	98.43

**Table 5:** Ablation study results on the specific components of the proposed architecture.

Model	Sensitivity (%)	Specificity (%)	Accuracy (%)
Only-GRU (No CNN)	88.26	96.67	90.84
Only-CNN (No Bi-GRU)	89.65	96.59	91.77
No KAN (Standard MLP)	98.62	93.82	97.15
Full	98.86	97.46	98.43

**Table 6:** Single dataset evaluation.

Dataset	Sensitivity (%)	Specificity (%)	Accuracy (%)
Ju	94.72	99.51	95.56
Ga	95.72	92.65	94.70
Si	98.83	96.70	97.87

**Table 7:** Cross-dataset evaluation.

Dataset	Sensitivity (%)	Specificity (%)	Accuracy (%)
LODO_Ju	98.41	97.49	98.13
LODO_Ga	98.33	97.04	97.93
LODO_Si	98.40	98.20	98.34

**Table 8:** Performance comparison of severity classification.

Methods	Scoring Scale	Accuracy (%)
Conv1D-Transformer [42]	H&Y Scale	87.89
DNN [17]	UPDRS	85.30
ANN [18]	H&Y Scale	87.10
SVM-RBF [43]	H&Y Scale	75.60
Static-Dynamic Net [23]	UPDRS	92.30
Proposed method	UPDRS	93.46

## Data Availability

We used public datasets as described and referenced in the main text.
